# Postmortem CT in decedents with SARS-CoV-2 infection. A single institution experience

**DOI:** 10.1080/20961790.2021.1977479

**Published:** 2022-01-24

**Authors:** Mariam Thomas, Fereidoun Abtin, Antoinette Roth, Catherine Yim, Anokh Pahwa, Jeremy Paige, Odey Ukpo

**Affiliations:** a Radiology, UCLA Medical Center Olive View, Los Angeles, CA, USA; b University of California Los Angeles, Los Angeles, CA, USA; c Los Angeles Medical Examiner-Coroner’s Office, Los Angeles, CA, USA

**Keywords:** Forensic sciences, postmortem CT, COVID-19, virtual autopsy, SARS-CoV-2

## Abstract

Coronavirus disease 2019 (COVID-19) caused by SARS-CoV-2 produced a global pandemic with significant mortality. As autopsies are not routinely performed on all decedents with SARS-CoV-2 infection, postmortem CT (PMCT) may be valuable to provide additional information on the cause of death and risk factors known to be associated with an increased mortality in COVID-19. The purpose of this manuscript is to review the PMCT findings in a series of 42 decedents with SARS-CoV-2 infection from our institution. Retrospective analysis of 42 decedents who had a positive postmortem nasopharyngeal swab for SARS-CoV-2 and had a PMCT were included in this study. Images were reviewed for pulmonary findings seen in COVID-19 and other organ involvement. Of the 42 decedents, although the majority had imaging findings in the lungs that would be consistent with COVID-19 and acute respiratory distress syndrome, in 14% of the decedents the SARS-CoV-2 infection was likely coincidental and the PMCT findings suggested that they died from other pathology. Over half of the decedents that died from COVID-19 had PMCT findings of vascular disease. PMCT is useful to identify pulmonary and extra pulmonary findings in decedents with SARS-CoV-2 infection that can provide additional information, which may be useful for the forensic pathologist to help determine the underlying cause of death.

Supplemental data for this article are available online at

## Introduction

Coronavirus disease 2019 (COVID-19) caused by severe acute respiratory syndrome coronavirus 2 (SARS-CoV-2) became a global pandemic in March 2020 [1]. As of July 5, 2021, globally, more than 184 million people were affected with the disease with nearly 4 million deaths [2]. As of July 4, 2021, over 600 000 people died from COVID-19 with Los Angeles County accounting for the greatest number of deaths in the US [2]. Given the large volume of cases and the risk of performing routine autopsies on COVID-19 patients, an alternative approach to gathering information regarding the death is needed. Postmortem CT (PMCT) is known to provide significant information with regards to specific forensic questions to determine the manner and cause of death in other disease pathologies and aids the forensic pathologist to determine whether a full autopsy is warranted [3]. PMCT has been proposed to be a useful screening tool to identify COVID-19 related fatalities in the medicolegal setting as well as has proven useful as triage tool prior to autopsy to identify possible COVID-19 examination, but there is a paucity of data regarding the PMCT findings of decedents with SARS-CoV-2 infection in the US [4, 5]. The purpose of this study is to document the PMCT findings in 42 decedents with SARS-CoV-2 infection at our institution in Los Angeles County.

## Methods

The study was presented for review to the Olive View-UCLA Education and Research Institute- Institutional Review Board. The Institutional Review Board in the US is the governing body for the institution which oversees and regulates research. The study was exempt from review.

Between March 1, 2020–December 12, 2020, 6 656 decedents were brought to the Los Angeles County Department of Medical Examiner-Coroner’s Office. The Los Angeles County Department of Medical Examiner-Coroner is mandated by law to determine and investigate the manner and cause of death in all violent, sudden, or unusual deaths occurring within Los Angeles County including all homicides, suicides, accidental deaths but also natural deaths where the deceased had not seen a physician within 20 days prior to the death. The Medical Examiner-Coroner’s Office can direct the forensic pathologist to perform autopsies and perform PMCT if necessary for the investigation of death.

Retrospective review was performed of all decedents from March 1, 2020–December 12, 2020, that while being evaluated by the forensic pathologist had a PMCT and had a positive postmortem test for SARS-CoV-2. Forty-two decedents were included for review and all decedents had positive postmortem nasopharyngeal swab test SARS-CoV-2 confirmed by real time reverse transcriptase polymerase chain reaction (RT-PCR). The RT-PCR testing and PMCT examinations had been performed within 24 h of when the deceased arrived at the Department of Medical Examiner-Coroner’s Office. The request for the RT-PCR and PMCT were made by the forensic pathologist after evaluation of the clinical history and the unknown manner of cause of death and to investigate whether the cause of death was due to COVID-19.

The decedents were scanned on a Phillips Brilliance 16 slice CT scanner (Philips Medical Systems, Madison, WI, USA) and images through the chest had been obtained without contrast on all cases. Image parameters for the chest was 120 − 140 kV, 150–300 mAs, 3 mm thickness. Additional imaging of the body had been obtained depending upon the individual clinical history by the forensic pathologist.

The PMCT images were evaluated retrospectively by three Board Certified Radiologist (CY, MT, AP) with 15, 15, and 10 years of radiology experience, respectively, and all with 3 years of PMCT experience. The radiologists were blinded to the official death record by the Department of Medical Examiner-Coroner’s Office. The PMCT images were reviewed initially individually and then consensus was obtained for the final interpretation.

The PMCT images were reviewed using a viewing console (Nil Read; Hyland Software, Ontario, Canada). The PMCT images were evaluated for (1) pulmonary findings reported in the radiology literature known to be associated with COVID-19 infection including bronchial wall thickening, air bronchograms, heterogeneous ground glass opacification, crazy paving, and consolidation [1, 6]; (2) atypical pulmonary findings not routinely seen with COVID-19 such as abscesses, cavitation and tree in bud appearance; (3) pleural effusions; (4) cardiovascular findings such as pericardial effusions, cardiomegaly, coronary artery calcifications and other vascular calcifications; as well as (5) general other organ appearance.

Clinical history was obtained by the coroner investigators who are trained to gather information regarding the circumstances leading to death and pertinent medical history. The decedents’ race was noted. This was determined by the physical appearance, family members and how the decedent identified themselves. Height and weight were recorded, and body mass index was calculated. A body mass index of greater than 25 was considered overweight, over 30 was considered obese and over 40 was morbidly obese.

## Results

A total of 42 deceased that had a PMCT, and postmortem tested positive for SARS-CoV-2 were included in the study, 34 males and 8 females, with mean age 49.6 years (range: 19 − 89 years). Race or ethnicity were Hispanic (78.5%, *n* = 33), White (11.9%, *n* = 5), African American (4.8%, *n* = 2), Asian (2.4%, *n* = 1) and Native American (2.4%, *n* = 1). Majority (90.5%, *n* = 38) of the cases were either overweight (26.2%, *n* = 11), obese (35.7%, *n* = 15) or morbidly obese (28.6%, *n* = 12) (Supplementary Table S1).

**Figure 1.**
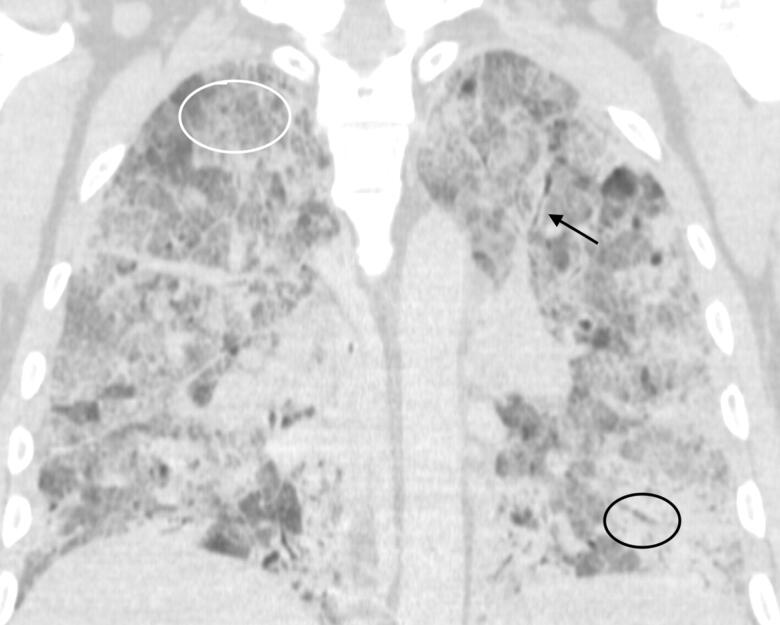
Postmortem CT (PMCT) coronal chest: one 54-year-old male with fever and cough with positive test for SARS-CoV-2. Pulmonary findings of COVID-19 in the deceased are shown with bronchial wall thickening (black arrow), air bronchograms as depicted as visible air within the bronchi surrounded by dense consolidated lung parenchyma (black circle) and a background of ground glass opacification as hazy appearance without underlying obscuration of the bronchi and vessels (white circle).

**Figure 2.**
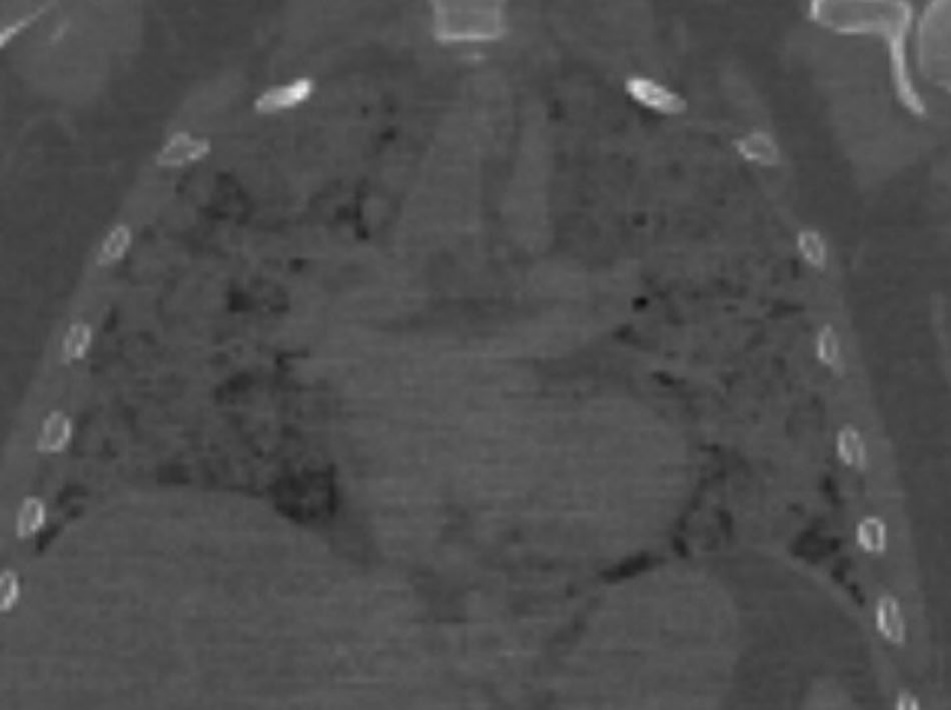
Postmortem CT (PMCT) coronal chest: one 38-year-old male complaining of cough, fever and difficulty breathing for 1 week. He had a negative test for SARS-CoV-2 the week prior to death. There are heterogenous areas of ground glass opacification and areas of consolidation throughout all lobes of both lungs. Although the deceased had a negative test for SARS-CoV-2 prior to death, the PMCT findings with the clinical history was suggestive of COVID-19 and postmortem testing was subsequently positive for SARS-CoV-2.

The majority (*n* = 35) of the deceased in which the official death certificate recorded the immediate cause of death from COVID-19 had pulmonary findings on the PMCT that would be consistent with COVID-19 (Supplementary Table S2). The spectrum of the pulmonary findings in these deceased ranged from heterogeneous ground glass opacifications and/or consolidation and crazy paving with bronchial wall thickening, air bronchograms, air filled spaces to complete consolidation of both lungs (Figures 1 and 2). One of the decedents had findings of PMCT evidence of superimposed bacterial infection with pulmonary abscesses.

**Figure 3.**
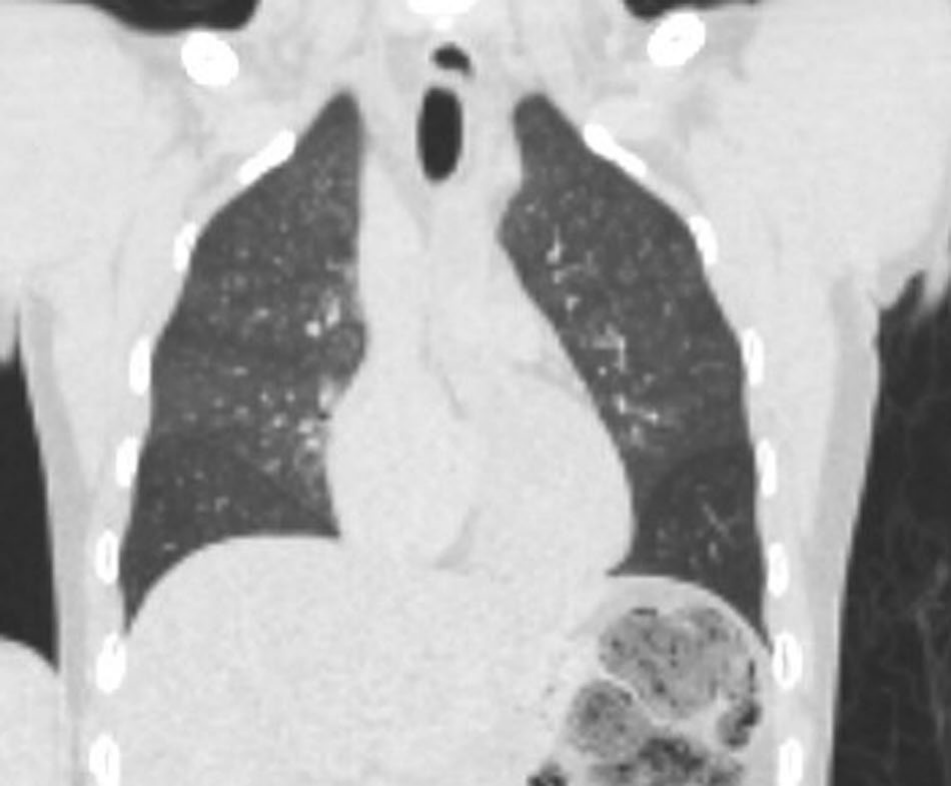
Postmortem CT (PMCT) coronal chest: one 30-year-old male with complaints of dizziness and nausea. He was found unresponsive in bed. Postmortem SARS-CoV-2 was positive, but the lungs are clear of consolidation or pulmonary findings of acute respiratory distress syndrome (ARDS). The primary cause of death on the official record was drug overdose due to fentanyl toxicity.

A small set of deceased (*n* = 3) had non-pulmonary findings on the PMCT that may have contributed to the death (one acute subdural hematoma, one dilated cardiomyopathy, and one intraparenchymal intracranial hematoma). A small set of deceased (*n* = 3) lacked the pulmonary findings often seen in COVID-19 on the PMCT. The official death record determined primary cause of death as drug toxicity in two of the cases (Figure 3) and hypertensive heart disease in the third. In all six of these cases in which the PMCT suggested that death was not due to COVID-19, the official immediate cause of death determined by the coroner was also recorded as not COVID-19. Alternatively, one decedent had PMCT findings that were consistent with COVID-19, and although the patient had recently been treated for COVID-19 pneumonia, the official primary cause of death was determined as acute bronchopneumonia and Klebsiella aerogenes infection with other significant condition as treated COVID-19.

Of the 35 decedents in which the official immediate cause of death was COVID-19, vascular disease with calcifications in the vasculature was identified in over half of the deceased (57%, *n* = 20). In approximately 11% (*n* = 4) of the cases the heart was enlarged, and 20% (*n* = 7) had pericardial effusions. In two cases, pleural effusions could not be determined due to the complete opacification of the chest. In the remaining 33, approximately 33% (*n* = 11) had pleural effusions.

## Discussion

The majority of our decedents had pulmonary findings on PMCT consistent with the COVID-19 diagnosis. This was characterized by bronchial wall thickening, heterogeneous ground glass opacities, crazy paving, and areas of consolidation [1, 6]. This is consistent with prior reports of PMCT in COVID-19 [4, 5, 7–9]. Although some studies on the autopsy findings in COVID-19 describe corresponding PMCT findings, there is a paucity of studies on PMCT with COVID-19, particularly in the US [9]. To date, the largest study on PMCT in COVID-19 is from O’Donnell et al. [5] in which 39 deceased who were referred for medicolegal investigation with positive SARS-CoV-2 RT-PCR testing also underwent a PMCT. They were evaluating the utility of PMCT as a screening test for COVID-19. Although the authors found that the PMCT may hint at COVID-19, they felt that nasopharyngeal swabs were needed for definitive diagnosis. Some of the PMCT findings they thought were extremely subtle or obscured by agonal changes. O’Donnell et al. [5] did believe that although PMCT alone should not be recommended as a tool for determining cause and manner of death due to COVID-19, PMCT could be helpful as a screening tool for COVID-19 if the clinical circumstances were appropriate. Although O’Donnell et al. [5] found some cases in which the PMCT findings were extremely subtle or obscured, in our study all the deceased with COVID-19 had extensive pulmonary findings seen diffusely throughout both lungs. Our findings are consistent with the prior study by De-Giorgio et al. [10] in which they reviewed the PMCT findings in 24 deceased and found that 100% of the deceased with COVID-19 had ground glass opacification on the PMCT. De-Giorgio et al. [10] felt that PMCT could be considered a reliable and safe modality to confirm COVID-19 pneumonia. Helmrich et al. [4] reviewed PMCT findings in 14 decedents with COVID-19 and showed that the predominant imaging findings were bilateral mixed densities, in either a diffuse or peripheral distribution, with traction bronchiectasis, and/or crazy paving. Helmrich et al. [4] found that traction bronchiectasis, ill-defined rounded consolidations, and reverse halo sign were useful when distinguishing from other postmortem changes, that PCMT could aid the forensic pathologist in diagnosing possible COVID-19 infection prior to autopsy, and that the PMCT could be useful as a triage tool. Williams et al. [7] also found in their study of five deceased with COVID-19, the PMCT findings included ground glass opacifications, crazy paving appearance and variable areas of more dense consolidation with relatively few areas of spared lung parenchyma. Williams et al. [7] also proposed the routine use of PMCT as a potential screening tool for the identification of COVID-19 related fatalities in the medicolegal system. In our study, all decedents who died from COVID-19 had ground glass opacification with consolidation, but this was in a diffuse pattern in all cases. There are several other smaller case reports and series all demonstrating the findings of COVID-19 with ground glass opacification and consolidation [8, 9, 11].

Although prior studies have reported that pleural effusions and mediastinal nodes are rare in COVID-19, Helmrich et al. [4] found that 36% of their cases had pleural effusions and 29% had mild lymphadenopathy. Our study showed similar findings, i.e., at least 33% of our deceased had pleural effusions and 36% had lymphadenopathy.

Many of the prior studies proved that PMCT could be useful as a screening tool to identify COVID-19. We also found that PMCT could be useful to identify additional pathology in decedents who were positive for COVID-19 but may have died from alternate pathology. In fact, six of the 42 cases (14.3%) testing positive for SARS-CoV-2, PMCT in conjunction with the clinical history and external examination suggested that the immediate death was not due to COVID-19. Although our study sample is small, in our population with approximately 14% of SARS-CoV-2-postitive death, the COVID-19 diagnosis may be coincidental and not the cause of death. This further substantiates that PMCT can be a useful triage tool to determine if the death was due to COVID-19.

In our study, we found that PMCT can provide useful information that can aid the forensic pathologist in determining whether there were additional contributing factors as to the immediate cause of death. In our study, not all deceased with COVID-19 appeared to have died from the disease. This is important as an accurate mortality rate from COVID-19 is critical to truly understand the disease burden. It is known that some individuals that test positive for the virus may be asymptomatic [12]. Decedents who were asymptomatic but SARS-CoV-2-positive and died from another cause should not be attributed to the death toll of COVID-19. The opposite is true as well as the PMCT can potentially identify decedents who truly did die from ARDS and COVID-19 but had false negative SARS-CoV-2 testing. One of our decedents had pulmonary findings on the PMCT and had died from COVID-19 but had a negative test for SARS-CoV-2 prior to death. This is not surprising as it is already known that SARS-CoV-2-negative test does not exclude disease [13]. Deceased who were false negative by testing but died from COVID-19 may be inaccurately excluded from the mortality statistics for COVID-19. Inaccuracies in the diagnostic tests for the virus including false negative results also undermine the consequences of the pandemic [14]. For the decedent’s families or clinicians who strongly suspect death from COVID-19 but have negative SARS-CoV-2 tests, PMCT can be offered as a non-invasive method for further clarification.

In our study, we found the pulmonary findings on PMCT in decedents who died from COVID-19 were similar to the findings on the limited number of autopsies that have been performed [15–19]. However, the cardiovascular findings did vary compared to prior reports. In the study by Fox et al. [17], examination of the heart was performed in nine autopsies on African American patients with COVID-19 which documented cardiomegaly and right ventricular dilatation in all the deceased. In a study by Elsoukkary et al. [19], autopsies were performed in 32 patients with COVID-19 and the heart was examined in 30 decedents and mild cardiomegaly was identified in 33% of their autopsies. This contrasts with our cases where only 11% of our deceased in which the immediate cause of death was COVID-19, had cardiac enlargement visible by PMCT. In the study by Fox et al. [17], autopsies did not show significant thrombosis of the coronary arteries or stenosis. However, Elsoukkary et al. [19] did find that over 50% of their decedents had at least moderate coronary artery atherosclerosis. In our study, we also found that over 50% of cases had evidence of vascular disease denoted by calcifications in the vasculature. It is possible that the cases in our study had pre-existing heart disease contributing to the cause of death as pre-existing heart disease is a known risk factor for increased mortality from COVID-19 [20].

Over 90% of the decedents in our study were either overweight, obese, or morbidly obese. This is consistent with reports that obesity is an independent risk factor for a poorer prognosis of COVID-19. Possible mechanisms attributed to the severity occur through higher ACE-2 concentrations, chronic inflammation and functional restrictive capacity in obese lungs [21]. In contrast to our study population, according to the Centers for Disease Control and Prevention, only 24.3% of adults in Los Angeles County are obese [22].

The majority of our decedents with COVID-19 were Hispanic. This is consistent with Los Angeles County public health data as of July 4, 2021, in which the age adjusted death rates due to COVID-19 per 100 000 was greatest in Hispanic/Latino with a mortality rate of 364 per 100 000. This is in contrast to Asians at 162, African Americans at 211 and Whites at 124 per 100 000 [23]. There are studies to evaluate these racial disparities with suggestions such as underlying health conditions, income discrepancies, access to health care, living circumstances and employment as factors [24]. According to the Los Angeles County Public Health Estimates as of July 2019, the population of Los Angeles County is 48.7% Hispanic, 27.9% White, 14.5% Asian, 8.5% Black, 0.2% Pacific Islander and 0.2% American Indian [25].

The study is limited as there are only 42 cases for review, but to our knowledge, this is the largest cohort of PMCT in deceased who were SARS-CoV-2 positive. We believe that PMCT is a valuable tool in deceased with COVID-19. The data obtained through PMCT would otherwise be lost due to the current perceived risk of performing traditional autopsies in patients who are positive for SARS-CoV-2. As we try to gather meaningful data on the disease, we believe that PMCT can be a valuable tool in this process.

## Supplementary Material

Supplemental Material
